# Screening and Awareness for Blood Pressure in a Non-Medical Setting: The Vienna Hairdresser Initiative

**DOI:** 10.3390/jcm14165639

**Published:** 2025-08-09

**Authors:** Simone Aufhauser, Michael Weber, Thomas W. Weiss, Maximilian Will

**Affiliations:** 1 Division of Internal Medicine 3, University Hospital St. Pölten, 3100 St. Pölten, Austria; 2 Department of General Health Studies, Karl Landsteiner University of Health Sciences, 3500 Krems, Austria; 3 Division of Biostatistics and Data Science, Department of General Health Studies, Karl Landsteiner University of Health Sciences, 3500 Krems, Austria; 4 Institute for Cardiometabolic Diseases, Karl Landsteiner Society, 3100 St. Pölten, Austria; 5 Medical School, Sigmund Freud University, 1010 Vienna, Austria

**Keywords:** arterial hypertension, awareness, primary prevention, non-medical setting, hairdresser

## Abstract

**Background:** Arterial hypertension (HTN) is the leading modifiable cardiovascular risk factor for overall mortality worldwide. In Austria, 1.6 million individuals above the age of 15, representing 20% of the total population and 70% of adults aged 65 and older, suffer from HTN. Despite numerous antihypertensive treatment options on the European market, only 38.8% of patients on optimal medical treatment (OMT) reach their treatment goal. Primary prevention remains a challenge, particularly for HTN and the consequential risk of cardiovascular diseases (CVDs). Thus, there is an urgent need for Disease Management Programs (DMPs). We sought to study a possible trial to diagnose hypertension in a non-medical setting at a very early stage of the disease and raise awareness for hypertension in affected people to avoid future complications of unrecognized and untreated HTN. For a non-medical setting, hairdressers fulfil many criteria for an optimal blood pressure (BP) measurement. **Methods:** This is a pilot study. A total of 193 individuals were included at a Viennese hairdresser. Metric data were described either using mean ± SD given normal distribution or median otherwise. Categorical data were described using absolute frequencies and percentages. For comparison, either independent *t*-tests or Mann–Whitney U tests or chi^2^ tests were assessed. The staff received expert training on how to measure blood pressure in a guideline-compliant way. All members signed the written and informed consent and received a questionnaire about their demographic data and cardiovascular risk factors. **Results:** Of the 193 participants in this study, 56.5% (109/193) were female and 43.5% (84/193) were male. The mean age was 54 ± 15.1 years. In the automatically measured office blood pressure (AOBP) measurement, the mean systolic BP was 137.1 ± 17.8 and the mean diastolic BP was 91.6 ± 11.2. Of all participants, 65.8% (127/193) were hypertensive, whereof 74.8% (95/127) had no treatment at all. Among 127 individuals evaluated, 63% (80/127) were unaware of their elevated blood pressure levels, while 28% (44/127) had a prior diagnosis of HTN. The control rate of the individuals with previously diagnosed HTN was very low, with only 18.5% [10.4; 30.9] reaching normotensive values in the current measurement. There was no difference in BP values of patients with previously diagnosed HTN and patients who were unaware of their disease. Antihypertensive treatment was being received by 20.2% (39/193), while 62.2% had not taken their prescribed blood pressure medication on the day of recruitment. **Conclusions:** This is the first Austrian study to show that screening for HTN in an unconventional non-medical setting is effective to diagnose HTN and raise awareness. Based on the even-higher-than-expected prevalence of HTN, we plan to conduct a cohort study in Vienna, inviting all hairdressers in socially deprived districts to act as gate openers for hypertensive subjects to raise awareness and to contact a regional GP for provision of medical care. An implementation of such a cost-effective and feasible disease management program in Austria might therefore reduce the burden of preventable cardiovascular events associated with HTN.

## 1. Introduction

Arterial hypertension (HTN) is the most important preventable cardiovascular risk factor for overall mortality worldwide [1]. In Austria, 1.6 million individuals above the age of 15 suffer from HTN [2]. Only 41% of patients with HTN in Austria have their HTN controlled [3].

A study enclosing twelve European countries (Austria, Belgium, France, Germany, Greece, Norway, Russia, Spain, Sweden, Switzerland, Turkey, and the United Kingdom) showed that on average only 38.8% of patients on optimal medical treatment (OMT) reach the target values, whereas 72.7% of 7642 patients ≥50 years suffer from HTN in these countries included in the trial [4].

Some trials in Canada and France have shown great success in establishing Disease Management Programs (DMPs) involving non-medical settings. The Canadian Hypertension Education Program (CHEP) resulted in a 106% increase in antihypertensive prescriptions between 1996 and 2006 and led to a >70% rate of patients reaching their target BP values [5]. In a prospective, multi-centre study, J.M. Boivin et al. demonstrated the effectiveness of screening programs in barber shops, demonstrating 75% of 1325 recruited persons showing hypertensive BP values [6].

The ‘China Rural Hypertension Control Project’ showed an increase from a 19.9% to a 57% control rate in a recent trial randomising 326 villages [7].

In Austria, the issue of uncontrolled HTN is worse than that in comparable European countries in terms of wealth and GDP. In 2003 the SCREEN II study demonstrated that only 17% reached their BP despite being on medical treatment [8,9]. Likewise, only 36% of Austrian study members reached their target BP values in EURIKA 2010 [4]. In a recent multi-centre study conducted in pharmacies in Lower Austria, we could show that from 4303 enrolled patients with HTN on medical treatment, only 41% reached their BP target [8]. Similar results were shown in the publication of the Austrian data of the “May Measurement Month” in 2017. Of 2711 persons who obtained a 3-on-1 AOBP measurement, 62.9% of all comers had hypertensive BP values (>140/90 mmHg), among whom 32.2% had hypertensive BP values (>140/90 mmHg) without prior established antihypertensive medication, and 63.5% of patients on medical treatment had hypertensive BP values. These results underline the importance of consistent BP follow-up screenings [8]. Furthermore, APOTHECARE and Herz Leben have shown similar findings [3,10].

HTN in its asymptomatic nature in the early stage of the disease leads to long-term issues, such as atherosclerosis resulting in end organ damage with severe secondary consequences such as stroke, coronary artery disease, heart failure, renal insufficiency, and peripheral artery disease [8,11].

Large epidemiologic studies have reported the lowest cardiovascular risk to be associated with systolic values < 115 mmHg and diastolic values < 75 mmHg [8].

However, there is a lack of data concerning the BP evaluation in non-medical settings. The findings of the previous Austrian studies showed that the majority of patients already diagnosed with HTN are not reaching their treatment target. There is, however, no Austrian data at all about individuals who are not seeking medical help. The Vienna hairdresser initiative is the first Austrian study to investigate screening, awareness, and control of HTN in an unconventional non-medical setting. A trial conducted at a hairdresser fulfils all the criteria for guideline-conforming BP measurement. With at least five minutes of comfortable sitting before starting, with an appropriately sized bladder cuff positioned at the level of the heart and repetitive measurements, a state-of-the-art BP measurement is possible [12].

As shown above, primary prevention remains a challenge in HTN, and due to the risk of developing cardiovascular diseases (CVD), there is an urgent need for Disease Management Programs (DMPs).

The primary objective of this pilot study was to study the feasibility of conducting a large cohort trial in an unconventional non-medical setting, investigating the prevalence of hypertensive patients, and as a secondary endpoint, the prevalence of insufficiently pre-treated HTN, meaning patients on medication with poor BP control, the analysis of awareness of HTN, and the correlations between HTN and the presence of cardiovascular risk factors.

## 2. Methods

### 2.1. Design

The Vienna hairdresser initiative is a pilot study to diagnose HTN in a non-medical setting at a very early stage of the disease. In this study, a very early stage is not associated with the age of the patient or the clinical staging, rather with the fact that the patient has no prior knowledge of their asymptomatic HTN. This achievement could lead to raising awareness for HTN in affected people and therefore avoid end organ damage. Furthermore, early diagnosis could support the accessibility to medical specialists.

For a non-medical setting, a hairdresser accomplishes all criteria for an optimal BP measurement, such as five minutes of comfortably sitting in an upright position [12]. Coiffeur LAKIM (Vienna, Austria) had no specific inclusion criteria. The hairdresser is located in the 17th district of Vienna (Austria). Socioeconomically, the 17th district shows a slightly lower average income and a lower overall educational level in comparison to that of the Viennese average [13]. The hairdresser asked all customers aged >18 years for participation in the study [14].

The Commission for Scientific Integrity and Ethics voted positive with respect to the ethical guidelines of the 1975 Declaration of Helsinki as reflected in a priori approval.

### 2.2. Study Population

The presumable sample size was 200 retrospectively available participants. From June to September 2021, 193 people participated in this feasibility study. Individuals aged 18 years or older were eligible for inclusion and provided written informed consent. The exclusion criterion was an age below 18 years.

### 2.3. Blood Pressure Measurements

The hairdresser was endued with an automatic sphygmomanometer device, the BOSO medicus uno (Bosch und Sohn, Jungingen, Germany). The staff received expert training on state-of-the-art blood pressure measurement. The measurement was performed on the naked left arm, with at least five minutes of comfortable sitting before starting blood pressure measurement and with an appropriately sized bladder cuff positioned at the level of the heart [12].

### 2.4. Questionnaire

All members of the study received a questionnaire about their demographic data, such as gender, age, educational qualification, occupation, relationship status, and postcode. Assessments on cardiovascular risk factors such as size, weight, smoking behaviour, presence of diabetes, hyperlipidaemia, heart failure or arterial HTN, family history, and medication were carried out. One part of the questionnaire requested information on who is treating their HTN and where participants get their information from.

### 2.5. Outcome

The primary endpoint of this study is an automatically measured office blood pressure (AOBP) defined as SBP ≥ 140 mmHg or DBP ≥ 90 mmHg in participants with insufficiently pre-treated HTN and those who are not aware of their status. The secondary endpoint is the prevalence of insufficiently pre-treated HTN, the analyzation of awareness of HTN, and the correlations between HTN and the presence of cardiovascular risk factors.

### 2.6. Statistical Methods

All statistical computations were performed using IBM SPSS Statistics for Windows version 27.0 (©IBM, Armon, NY, USA). Metric data are described either using mean ± SD given normal distribution or median (1st and 3rd quartile) otherwise. Categorical data are described using absolute frequencies and percentages. In order to compare awareness with education, relationship, profession, sex, smoking, diabetes mellitus, hypercholesterinaemia, heart failure, and family history, either independent *t*-tests (for metric and normally distributed data and in the case of homogenous variances), Welch-corrected *t*-tests (in the case of metric and normally distributed data but heterogenous variances), Mann–Whitney U tests (for metric but skewed data), or chi^2^ tests (given categorical data) were assessed. A *p*-value equal or below 0.05 was considered statistically significant.

## 3. Results

The recruiting lasted from the 25 June to the 30 September 2021. Due to COVID lockdowns and the consequent shutdowns for hairdressers, recruitment was postponed, and the sample size was reduced.

We enrolled 193 individuals, 109 (56.5%) female and 84 (43.5%) male persons with a mean age of 54 ± 15.1 years. The mean systolic BP was 137.1 ± 17.8 and the mean diastolic BP was 91.6 ± 11.2. Using an automatically measured office blood pressure (AOBP), defined as an SBP ≥ 140 mmHg or DBP ≥ 90 mmHg, 65.8% (127/193) of participants had hypertensive BP measurement values.

**Figure 1:**
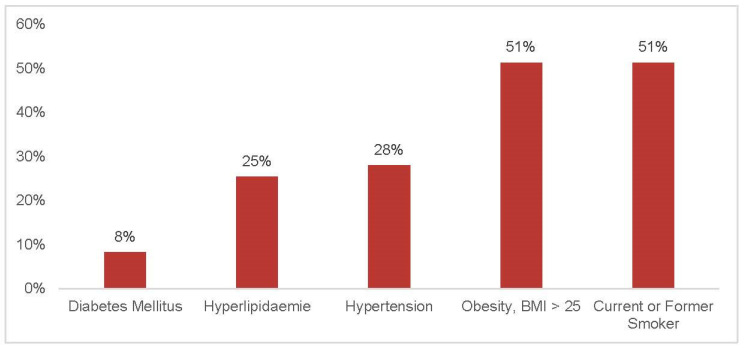
Distribution of cardiovascular risk factors (x-axis) in percent (y-axis).

**Figure 2:**
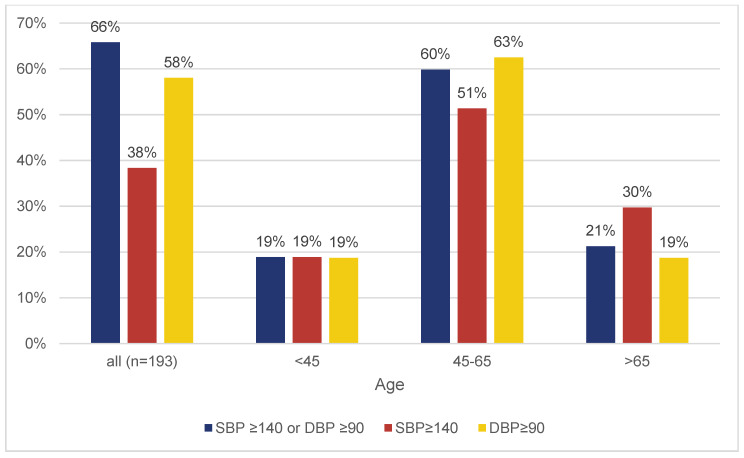
Distribution of hypertensive BP values, separated in age groups (x-axis) in percent (y-axis).

**Figure 3:**
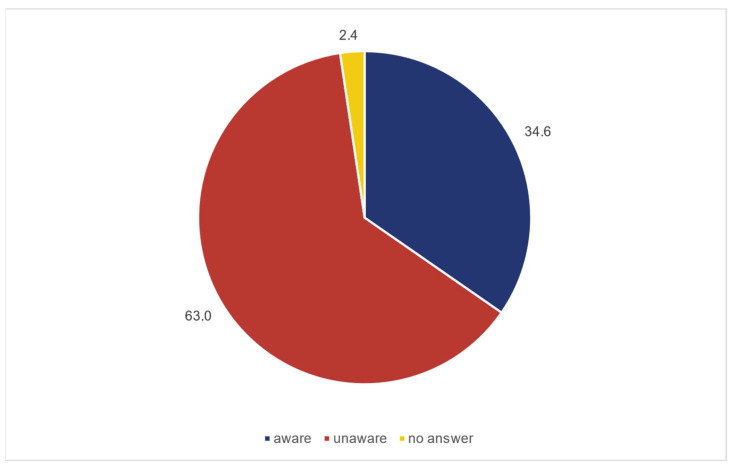
Distribution of the awareness of hypertension in percent.

Among the 193 individuals, 28% (54/193) were already diagnosed with HTN, of whom 18.5% [10.4; 30.9] exhibited normotensive values in the current measurement (Figure 1). Of the 127 participants with elevated blood pressure measurements, 63% (80/127) were unaware of their condition (Figure 1). Figure 2 indicates that the cohort of participants below 45 years of age have with 19 percent the lowest rate of elevated blood pressure. Additionally, 60.1% (116/193) were aware of the high cardiovascular risk associated with HTN (Figure 3). 

Antihypertensive treatment was being received by 20.2% (39/193), while 62.2% had not taken their prescribed blood pressure medication on the day of recruitment.

With respect to cardiovascular risk factors other than HTN, 51.3% (99/193) were current or former smokers; the average BMI (kg/m^2^) in all individuals was 26 ± 4; 25.4% (49/193) suffered from hyperlipidaemia or took medication against high cholesterol; 8.3% (16/193) suffered from diabetes mellitus; and 4.1% (8/193) reported heart failure (Table 1). Hyperlipidaemia (*p* = 0.009) and heart failure (*p* = 0.038) were statistically significantly associated with HTN (Table 2 and Table 3).

There is a statistically significant correlation between diabetes mellitus (*p* = 0.015), hyperlipidaemia (*p* < 0.001), heart failure (*p* < 0.001), and patients’ awareness.

Most participants (44%) did not answer the question “Who’s treating your HTN?” A total of 35.8% were in the hands of their GPs, and 17.1% were treated by internal specialists or cardiologists.

Interestingly, there was no difference in BP values of patients with previously diagnosed HTN and patients who were unaware of their disease. If information about HTN was gathered, participants mostly obtained it from their GP (41.5%), followed by family and friends (10.9%), or newspapers and magazines (9.8%). A total of 27.5% did not gather information about HTN at all.

Further sociodemographic data and clinical characteristics are presented in Table 1. Table 4 shows the logistic regression of all independent variables and the dependent variable (BP). As there were eight participants with heart failure, of which seven had HTN, a logistic regression was not suitable. The logistic regression shows a significant result for hyperlipidaemia.

## 4. Discussion

In this pilot study, we could show that 65.8% of participants had hypertensive BP values. Only 22.8% of the attendants with hypertensive values had already been on medical therapy, whereas the great majority (74.8%) were without treatment at all. According to our findings, it seems reasonable to assume that the estimated number of undetected cases is very high in comparable regions in Austria.

Given the significant correlation between diabetes mellitus, hyperlipidaemia, heart failure, and patients’ level of awareness, we conclude that individuals actively involved in the management of chronic diseases are more likely to recognize their hypertensive status and, consequently, achieve more effective blood pressure control.

The fact that there is no significant difference in BP values between patients with previously diagnosed HTN and patients who were unaware of their disease shows that blood pressure management should be strengthened. Moreover, 62.2% of the patients already diagnosed with HTN did not take their antihypertensive medication on the day of inclusion. This shows that there is a significant lack of awareness of the importance of the regular intake of medication. If this is due to poor adherence, lack of awareness needs further research.

Thus, there is an urgent need for action to implement of a cost-effective and feasible disease management program to reduce the burden of preventable cardiovascular events associated with HTN. Similar to the “Apothecare” trial, we therefore plan to conduct a cohort study in Vienna, inviting all hairdressers in socially deprived districts to act as gate openers for hypertensive subjects to raise awareness and refer individuals with newly detected HTN or undertreated HTN to a regional GP for provision of medical care.

## 5. Limitations

The sample size did not meet the initial prediction due to severe COVID lockdown restrictions for hairdressers. Further, there was no assessment of used stimulants (e.g., smoking) prior to entering the hairdresser. At least five minutes of comfortable sitting without the use of stimulating agents affecting blood pressure could be assured. Moreover, kidney diseases had not been assessed separately in the manner of cardiovascular risk factors. The data represent one cohort, which limits the generalizability. Further, we have no data on how many people declined participation; every client of the hairdresser was invited to join the study.

## 6. Conclusions

The Vienna hairdresser initiative is the first study of its kind in Austria. It confirms that screening for HTN at the hairdresser as an unconventional setting in deprived social areas seems to be an effective tool to detect HTN in previously unaware subjects. This could lead to earlier diagnosis and raising awareness for HTN in affected people and could thereby contribute to the avoidance of secondary diseases.

## Figures and Tables

**Table 1:** Baseline characteristics, socioeconomic factors, and co-morbidities in the trial population.

	Details	All Individuals
Age		54 ± 15
Gender	Female	56.5%
	Male	43.5%
BMI		26 ± 4
SBP ≥ 140 mmHg OR DBP ≥ 90 mmHg		65.8%
Systolic AOBP_1 (mmHg)		137.1 ± 17.8
Diastolic AOBP_1 (mmHg)		91.6 ± 11.2
HR_1 (bpm)		77.8 ± 11.4
Systolic AOBP_2 (mmHg)		134.4 ± 16.8
Diastolic AOBP_2 (mmHg)		90.2 ± 11.3
HR_2 (bpm)		76.8 ± 11.9
Marital Status	Single	24.4%
	Married or Partnership	54.4%
	Divorced	13.5%
	Widowed	5.7%
	n/a	2.1%
Education	Compulsory Education	12.4%
	Apprenticeship	38.9%
	School Leaving Examination	29.5%
	University Degree	16.6%
	n/a	2.6%
Occupation	Employed	53.9%
	Retired	28.5%
	Unemployed	4.7%
	Freelancer	7.8%
	n/a	5.2%
Smoking	Never	46.1%
	Prior	30.6%
	Current	20.7%
	n/a	2.1%
Diabetes Mellitus		8.3%
Hyperlipidaemia		25.4%
Heart Failure		4.1%
Family History, Stroke, or MCI < 50 y		19.7%
Hypertension in Anamnesis		28%
Knowledge about High CV Risk in Hypertension		60.1%
Medication against Hypertension		20.2%
Medication Taken Today?	Yes	20.2%
Who is Treating Your Hypertension?	GP	35.8%
	Internal Specialist	11.4%
	Cardiologist	5.7%
	Hospital	2.6%
	Others	7.8%
	n/a or not known	44.0%
Where Do You Get Information about Hypertension from?	No information obtained	27.5%
	Family and Friends	10.9%
	Newspaper/Magazines	9.8%
	Internet	12.4%
	Pharmacies	6.7%
	GP	41.5%
	n/a	11.4%

BMI: Body mass index, SBP: systolic blood pressure, DBP: diastolic blood pressure, AOBP: automatically measured office blood pressure, HR: heart rate, MCI: myocardial infarction, CV: cardiovascular, GP: general practitioner.

**Table 2:** Association between hyperlipidaemia and HTN.

*p* = 0.009			>140 and/or >90		Total
			Normal BP	>140 and/or >90	
**Hyperlipidaemia**	**yes**	n	9	40	49
		%	18.4%	81.6%	100.0%
	**no**	n	54	85	139
		%	38.8%	61.2%	100.0%
**Total**		n	63	125	188
		%	33.5%	66.5%	100.0%
**OR (95% CI)**			0.354 (0.159–0.788)		

**Table 3:** Association between Heart Failure and HTN.

			>140 and/or >90		Total
			Normal BP	>140 and/or >90	
**Heart Failure**	**yes**	n	0	8	8
		%	0.0%	100.0%	100.0%
	**no**	n	63	114	177
		%	35.6%	64.4%	100.0%
**Total**		n	63	122	185
		%	34.1%	65.9%	100.0%

**Table 4:** Logistic regression of all independent variables.

	B	SE	*p*-Value	OR	OR (95% CI)	
					Lower Limit	Upper Limit
**DM**	−0.095	0.748	0.899	0.910	0.210	3.941
**BMI**	0.037	0.042	0.377	1.038	0.956	1.126
**Family History/MCI/Stroke**	−0.476	0.457	0.297	0.621	0.254	1.521
**Smoking**	−0.138	0.206	0.501	0.871	0.582	1.303
**Hyperlipidaemia**	−0.925	0.458	0.043	0.396	0.162	0.972

## Data Availability

The original contributions presented in this study are included in the article. Further inquiries can be directed to the corresponding author.

## Abbreviations

AOBP	Automatically Measured Office Blood Pressure
BP	Blood Pressure
CI	Confidence Interval
CHEP	Canadian Hypertension Education Program
CVD	Cardiovascular Diseases
DBP	Diastolic Blood Pressure
DMP	Disease Management Program
ESC	European Society of Cardiology
GDP	Gross Domestic Product
GP	General Practitioner
HMBP	Home Monitoring of Blood Pressure
HTN	Arterial Hypertension
ISH	International Society of Hypertension
n/a.	Not available
OMT	Optimal Medical Therapy
OR	Odds Ratio
SBP	Systolic Blood Pressure
SD	Standard Deviation
